# Einflussfaktoren bei der Wahl der Androgendeprivationstherapie für Patienten mit hormonsensitiven Prostatakarzinom: Ergebnisse der ProComD-Studie

**DOI:** 10.1007/s00120-021-01620-7

**Published:** 2021-08-17

**Authors:** J. Lehmann, C. W. Kluike, A. Haider, K. S Haider, S. Baumann, M. Flesch, M. Gedamke, D. Kägebein

**Affiliations:** 1 Urologische Gemeinschaftspraxis Prüner Gang, Gesundheitszentrum Kiel-Mitte, Prüner Gang 15., 24103 Kiel, Deutschland; 2 Urologie am Wasserturm, Lüneburg, Deutschland; 3 Praxis für Urologie und Andrologie, Bremerhaven, Deutschland; 4 Praxisgemeinschaft für Urologie, Leipzig, Deutschland; 5 Marienkrankenhaus, Soest, Deutschland; 6 msc, Kiel, Deutschland; 7 Ferring Arzneimittel GmbH, Kiel, Deutschland

**Keywords:** Degarelix, GnRH-Antagonist, Therapieentscheidung, Komorbiditäten, Behandlungspfade, Degarelix, GnRH antagonist, Treatment decision, Comorbidities, Treatment options

## Abstract

**Hintergrund:**

Die Androgendeprivationstherapie (ADT) mit einem GnRH-Agonisten (Gonadotropin-releasing-Hormon) oder -Antagonisten stellt einen zentralen Bestandteil der Behandlung des Prostatakarzinoms (PCa) dar. Über die Faktoren, welche die Wahl der ADT beeinflussen, ist bis jetzt wenig bekannt.

**Ziele der Arbeit:**

Faktoren, welche die Wahl der ADT bei Patienten mit hormonsensitivem PCa beeinflussen, werden identifiziert. Vom Urologen zur Identifizierung von Begleiterkrankungen genutzte Informationsquellen sowie deren Prävalenzen werden bestimmt.

**Methoden:**

Die zweiarmige, prospektive, nicht-interventionelle Studie „ProComD“ wurde von Sept. 2014 bis Juni 2019 an 80 Studienzentren in Deutschland durchgeführt. Patienten mit hormonnaivem PCa und Notwendigkeit einer ADT wurden nach erfolgter Therapieentscheidung in die Studie eingeschlossen. Fragen bezüglich Informationsquelle und Therapieentscheidung wurden vom Arzt direkt im elektronischen Datenerfassungssystem (eCRF) beantwortet.

**Ergebnisse:**

Es wurden Daten von 413 Patienten ausgewertet (Degarelix *n* = 268; GnRH-Agonisten *n* = 145). Ausschlaggebend für die Therapieentscheidung waren für beide Behandlungsgruppen u. a. die Faktoren Komorbiditäten (bei 42 % aller Patienten), Compliance (64 %) und Alter (81 %).

Die häufigste konsultierte Informationsquelle bzgl. vorhandener Komorbiditäten ist die Anamnese durch den behandelnden Urologen selbst (68,5 % in beiden Gruppen). Bei Patienten mit kardiovaskulären Vorerkrankungen wurde zusätzlich der Arztbrief (45,8 % Degarelix vs. 38,9 % GnRH-Agonisten) oder der Anamnese-Fragebogen (38,9 % Degarelix vs. 20 % GnRH-Agonisten) herangezogen.

**Schlussfolgerungen:**

Komorbiditäten zählen neben dem Alter und der Compliance zu den wichtigen Faktoren, die die Wahl der ADT beeinflussen.

## Abstract (en)

**Background:**

Androgen deprivation therapy (ADT) with a GnRH agonist or the GnRH antagonist degarelix is a central component in the treatment of prostate cancer (PCa). Little is currently known regarding the decision criteria. Knowledge of these could improve the success of treatment in the future.

**Objectives:**

To identify factors influencing the treatment decision in patients with hormone-sensitive prostate cancer receiving ADT and to determine the incidence of concomitant disease in both treatment groups.

**Methods:**

The two-arm, prospective, non-interventional study “ProComD” was conducted from September 2014 to June 2019 at 80 study centers in Germany. After the therapy decision was made, patients with hormone-sensitive prostate cancer needing ADT were included in the study. Data were collected during routine visits.

**Results:**

Data from 413 patients were evaluated (degarelix *N* = 268; GnRH agonists *N* = 145). Key factors influencing the therapy decision for both treatment options included comorbidities (42% of all patients), compliance (64%), and age (81%). The source of information consulted most frequently regarding existing comorbidities was the patient’s medical history conducted by the treating urologist themselves (65% in both groups). For patients with pre-existing cardiovascular diseases, the doctor’s letter (45.8% degarelix vs. 38.9% GnRH agonists) or the medical history questionnaire (38.9% degarelix vs. 20% GnRH agonists) was additionally taken into account.

**Conclusion:**

Comorbidities along with age and compliance are among the key factors influencing the treatment decisions made by urologists.

Die Androgendeprivationstherapie (ADT) mit GnRH(Gonadotropin-Releasinghormon)-Agonisten oder dem GnRH-Antagonisten Degarelix ist seit Jahrzehnten der Behandlungsstandard beim fortgeschrittenen hormonnaiven Prostatakarzinom (PCa). Entscheidungskriterien für den Einsatz einer der beiden Substanzklassen wurden bislang nicht identifiziert. Diese Studie gibt Hinweise, wie Basischarakteristika und Komorbiditäten der PCa-Patienten die Therapieentscheidung beeinflussen.

## Hintergrund

In der Therapie des hormonnaiven PCa wird der medikamentöse Androgenentzug in der Regel entweder durch die Behandlung mit GnRH-Agonisten oder -Antagonisten erreicht [25]. Das *Outcome* der ADT wird stark von Komorbiditäten beeinflusst, und Beobachtungen deuten darauf hin, dass Patienten, die sich einer ADT unterziehen, einem erhöhten Risiko für behandlungsbedingte unerwünschte Ereignisse ausgesetzt sein können, insbesondere für kardiovaskuläre Ereignisse [7, 13, 17]. PCa-Patienten weisen aufgrund ihres meist höheren Alters häufig Komorbiditäten auf – insbesondere finden sich oft kardiovaskuläre Vorerkrankungen in ihrer Anamnese [5].

Randomisierte klinische Studien, Metaanalysen und Real-world-Evidenzstudien haben gezeigt, dass das Risiko kardiovaskulärer Ereignisse unter einer ADT bei Patienten, die mit einem GnRH-Antagonisten behandelt werden, im Vergleich zu den mit GnRH-Agonisten behandelten Patienten reduziert ist [1, 3, 8, 12, 16, 20, 21]. Dies impliziert ein unterschiedliches Risiko für kardiovaskuläre Ereignisse in Abhängigkeit der ADT mit GnRH-Agonisten oder -Antagonisten [18].

Die Kenntnis über bestehende Komorbiditäten der Patienten zum Zeitpunkt der Therapieentscheidung kann deshalb von wesentlicher Bedeutung für die effektive und sichere Behandlung der PCa-Patienten sein. Voraussetzung für die Kenntnis über Komorbiditäten ist eine hohe Qualität der Anamnese. Die Qualität der Anamnese, die Entscheidungskriterien für eine Therapie mit GnRH-Agonisten oder Degarelix sowie das Ausmaß der interdisziplinären Zusammenarbeit während der PCa-Behandlung sind jedoch bisher nicht bekannt.

### Ziel der Studie

Ziel der Studie war es, Faktoren zu identifizieren, welche bei Patienten mit hormonnaivem PCa die Therapieentscheidung bzgl. der medikamentösen ADT (Degarelix oder GnRH-Agonisten) beeinflussen. Darüber hinaus wurden die Häufigkeit von Begleiterkrankungen und die von den Urologen hierfür herangezogenen Informationsquellen bestimmt.

## Methodik

### Studiendesign

Die zweiarmige, prospektive, nicht-interventionelle Studie (NIS) ProComD wurde vom 17. September 2014 bis 30. Juni 2019 an 80 Studienpraxen in Deutschland durchgeführt. Die Patienten wurden mit der Verschreibung einer medikamentösen ADT (Degarelix oder GnRH-Agonist) unmittelbar in die Studie aufgenommen und bis zu 48 Monate lang nachbeobachtet. Die NIS wurde von der Ethikkommission der Landesärztekammer Nordrhein hinsichtlich berufsethischer und -rechtlicher Gesichtspunkte begutachtet (Berufsrechtliche Beratung nach § 15 der Berufsordnung). Die Patienten gaben nach Aufklärung schriftlich ihr Einverständnis, bevor sie an der Studie teilnahmen.

### Patienten und Behandlung

Patienten mit hormonnaivem PCa (lokal, lokal fortgeschritten oder metastasiert) und der Indikation einer ADT wurden nach der Therapieentscheidung des Arztes in die Studie eingeschlossen. Nicht erlaubt war der Einschluss von Patienten, die eine andere hormonelle Behandlung ihres PCa erhielten. Ausgenommen hiervon waren Patienten mit bereits erfolgter hormoneller neoadjuvanter oder adjuvanter Behandlung im Rahmen einer kurativ intendierten Primärtherapie, wenn diese Behandlung nicht länger als 6 Monate durchgeführt wurde und mindestens 6 Monate vor Studieneinschluss abgeschlossen war. Eine Mindestdauer der ADT war nicht vorgegeben.

Degarelix wurde als 1‑Monats-Formulierung mit einer Startdosis von 240 mg und einer Erhaltungsdosis von 80 mg verabreicht. Als GnRH-Agonisten wurden Leuprorelinacetat, Goserelinacetat, Buserelinacetat sowie Triptorelinacetat (jeweils als 1‑, 3‑ oder 6‑Monats-Fomulierung) eingesetzt. Diagnostik, Medikation und Patientenüberwachung lagen ausschließlich im Ermessen des Arztes entsprechend seiner üblichen Behandlungspraxis. Die Visiten einschließlich ihrer Dokumentation waren Teil der klinischen Routine und fanden nicht zu im Voraus definierten Zeitpunkten statt.

### Ziele

Primäres Ziel der Studie war es, Faktoren zu identifizieren, die zur Entscheidung für eine medikamentöse ADT bei Patienten mit hormonnaivem PCa mit und ohne Komorbiditäten beitragen. Folgende Aspekte wurden dabei insbesondere bewertet: 1) Kenntnisse des Urologen in Bezug auf die Krankengeschichte der Patienten, Begleiterkrankungen und Begleitmedikation sowie Risikofaktoren zum Zeitpunkt der Entscheidungsfindung sowie die hierfür herangezogenen Informationsquellen. 2) Basierend auf 1) sollte festgestellt werden, wie diese Kenntnisse die behandelnden Ärzte in ihrer Therapieentscheidung beeinflusst haben. Ein weiteres Ziel war die Bestimmung der Häufigkeiten verschiedener Komorbiditäten zu Therapiebeginn.

### Datenerhebung

Die Basischarakteristika wurden in Routinevisite 1 vom behandelnden Arzt erfragt oder aus der Krankenakte entnommen. Folgedaten in den weiteren Routinevisiten wurden alle 3 (± 4 Wochen) Monate für das erste Jahr und alle 6 Monate (± 6 Wochen) für die folgenden 3 Jahre erhoben. Die Daten wurden in ein elektronisches Datenerfassungssystem übertragen.

Fragen bezüglich der konsultierten Informationsquellen und der Faktoren, die die Therapieentscheidung beeinflussten, wurden vom Arzt ebenfalls direkt im elektronischen Datenerfassungssystem (eCRF) innerhalb eines Fragebogens beantwortet. Begleiterkrankungen wurden gemäß MedDRA (Vers. 19.0) und Begleitmedikation nach ATC-Code kodiert.

### Subgruppen

Folgende Subgruppen wurden definiert: Patienten mit/ohne Metastasen, Patienten mit einem Basis-PSA-Wert 0–50 ng/ml und > 50 ng/ml und Patienten ohne/mit kardiovaskulären Vorerkrankungen (MedDRA SOC „cardiac disorders“ und/oder „vascular disorders“).

### Statistik

Statistische Analysen wurden mittels SAS, Version 9.3 (SAS Institute Inc., Cary, NC, USA) durchgeführt. Die finalen Auswertungen wurden für die FAS-Population durchgeführt. Diese bestand aus allen Patienten, die für die jeweilige Behandlung eingeteilt wurden. Darüber hinaus musste die erste Routinevisite sowie mindestens eine weitere *Follow-up*-Visite dokumentiert sein. Patienten, die die ADT-Behandlung abbrachen, wurden für die Safety-Analysen weiterhin untersucht.

Soweit Vergleiche zwischen Behandlungsgruppen durchführt wurden, wurden diese als adjustierte Unterschiede (metrische Variablen) oder Raten (kategorische Variablen), jeweils mit einem Konfidenzintervall von 95 %, dargestellt. Die Vergleiche wurden hinsichtlich Unterschiede in den Baselineparameter mittels einer Regressionsanalyse adjustiert.

Um Korrelationen zwischen der Wahl der Therapie und vorbestehenden oder neuen Komorbiditäten zu untersuchen, wurde ein χ^2^-Test verwendet. Beim Auftreten von kleinen erwarteten Frequenzwerten wurde ein exakter Fisher-Test (ggf. unter Verwendung einer Monte-Carlo-Annäherung) angewandt. Um Unterschiede zwischen den Behandlungsgruppen zu untersuchen, wurde für metrische Variablen ein Student’s t‑Test oder Wilcoxon-Rangsummentest und für kategoriale Variablen ein χ^2^- (> 2 Kategorien) oder ein Binomialtest angewendet. Alle abgeleiteten *p*-Werte sind deskriptiv zu interpretieren. Das Signifikanzniveau wurde für alle angewandten Testverfahren auf 5 % festgelegt.

Die Studie benötigte keine bestätigenden statistischen Tests, da sie dazu angelegt war, die Qualität der Anamnese und die Effektivität und Sicherheit der Behandlung mit Degarelix und GnRH-Agonist zu berichten. Alle untersuchten Hypothesen waren zweiseitig. Für ordinalskalierte Korrelationen wurde der Spearman-Koeffizient verwendet, für metrische Korrelationen der Pearson-Koeffizient.

## Ergebnisse

### Demographie, Basisdaten und Subgruppen

Es konnten die Daten von 413 Patienten ausgewertet werden (Degarelix *n* = 268; GnRH-Agonisten *n* = 145). Folgende GnRH-Agonisten wurden zur Behandlung eingesetzt: Buserelin (11,1 %; *n* = 46), Leuprorelin (19,4 %; *n* = 80), Triptorelin (4,4 %; *n* = 18) und Goserelin (0,2 %; *n* = 1). Es wurden keine statistisch signifikanten und klinisch relevanten Unterschiede zwischen den Behandlungsgruppen hinsichtlich der Demographie und anderer Basischarakteristika beobachtet (Tab. 1).

**Table 1:** 

	Degarelix (*n* = 268)	GnRH-Agonisten (*n* = 145)	Gesamt (*n* = 413)
*Alter (Jahre)*			
Mittelwert ± SD	73,6 ± 8,3	74,9 ± 8,35	74,0 ± 8,34
Median	75	76	76
*Größe (m)*			
Mittelwert ± SD	1,75 ± 0,07	1,74 ± 0,07	1,75 ± 0,07
Median	1,75	1,74	1,75
*Gewicht (kg)*			
Mittelwert ± SD	82,9 ± 13,1	83,0 ± 15,8	82,9 ± 82,9
Median	82,0	82,0	82,0
*Body Mass Index (BMI)*			
Mittelwert ± SD	27,2 ± 4,0	27,2 ± 4,3	27,2 ± 4,1
Median	26,7	26,5	26,6
*Testosteron (ng/dl)*			
Mittelwert ± SD	385,4	359,2	378,1
Median	317,3	330,2	320,0
*PSA (ng/ml)*			
Mittelwert ± SD	129,0 ± 382,8	111,6 ± 309,5	122,7 ± 357,8
Median	21,1	9,8	15,3
Range	0–4565	0–2289	0–4565
*PSA-Untergruppen*			
< 10 ng/ml	91 (34,0 %)	69 (47,6 %)	160 (38,7 %)
10–20 ng/ml	28 (10,4 %)	16 (11,0 %)	44 (10,7 %)
< 20–50 ng/ml	43 (16,0 %)	16 (11,0 %)	59 (14,3 %)
> 50 ng/ml	82 (30,6 %)	36 (24,8 %)	118 (28,6 %)
*Gleason-Score*			
2–4	1 (0,4 %)	1 (0,7 %)	2 (0,5 %)
5–6	38 (14,2 %)	17 (11,7 %)	55 (13,3 %)
7	75 (28,0 %)	50 (34,5 %)	125 (30,3 %)
8–10	139 (51,9 %)	71 (49,0 %)	210 (50,8 %)
Keine Angabe	15 (5,6 %)	6 (4,1 %)	21 (5,1 %)
Mittelwert ± SD	7,8 ± 1,2	7,7 ± 1,2	7,7 ± 1,2
Median	8,0	8,0	8,0
*Krankheitsstadium*			
Lokal begrenzt (T_1–2_, N_0_/*N* _x_, M_0_)	50 (18,7 %)	46 (31,7 %)	96 (23,2 %)
Lokal fortgeschritten (T_3–4_, N_0_/*N* _x_, M_0_)	29 (10,8 %)	13 (9,0 %)	42 (10,2 %)
Fortgeschritten bzw. metastasiert (N_1_ und/oder M_1_)	92 (34,3 %)	40 (27,6 %)^a^	132 (32,0 %)
Nicht kategorisierbar (T_x_ und/oder M_x_)	97 (36,2 %)	46 (31,7 %)	143 (34,6 %)

^a^*p* < 0,0001, *p*-Werte basieren auf einem Binomialtest

Für 407 Patienten waren Daten zur Gesamtdauer der ADT innerhalb der Studie vorhanden. Die durchschnittliche Dauer der ADT betrug für die Degarelix-Patienten 22,4 Monate (SD = 13,7) und für die Patienten, die mit einem Agonisten behandelt wurden 27,0 Monate (SD = 13,9).

Das Krankheitsstadium bei Einschluss in die Studie war signifikant unterschiedlich zwischen den beiden Behandlungsgruppen (*p* = < 0,0001). In der Degarelix-Gruppe war das metastasierte PCa das am häufigsten dokumentierte Krankheitsstadium (34,3 %), während in der GnRH-Agonistengruppe das lokal begrenzte PCa am häufigsten dokumentiert wurde (31,7 %; Tab. 1).

Der prozentuale Anteil der Degarelix-Patienten war in den Subgruppen „Patienten mit kardiovaskulären Vorerkrankungen“ (66,8 vs. 62,1 %), „Patienten mit Baseline-PSA > 50 ng/ml“ (30,6 vs. 24,8 %) und „Patienten mit Metastasen“ (36,9 vs. 24,8 %) jeweils signifikant höher (*p* < 0,0001; Tab. 2).

**Table 2:** 

Subgruppe	Degarelix (*n* = 268)	GnRH-Agonisten (*n* = 145)	Gesamt (*n* = 413)
Metastasen M+	99 (36,9 %)	36 (24,8 %)^a^	135 (32,7 %)
PSA > 50 ng/ml	82 (30,6 %)	36 (24,8 %)^a^	118 (28,6 %)
Kardiovaskuläre Vorerkrankung(en) (inklusive Hypertonie)	179 (66,8 %)	90 (62,1 %)^a^	269 (65,1 %)

^a^*p* < 0,0001, p‑Werte basieren auf einem Binomialtest

### Komorbiditäten, Risikofaktoren, Begleitmedikation

**Figure 1:**
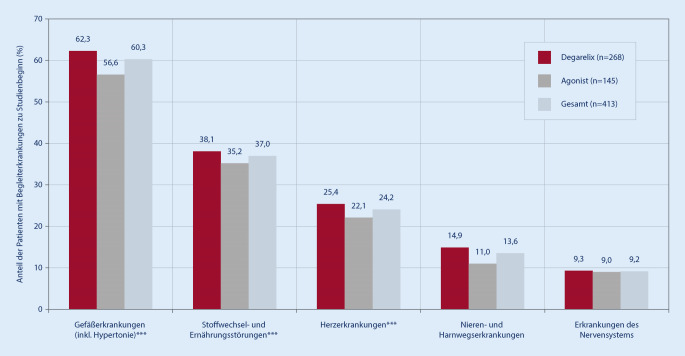

Insgesamt wurde bei 75,5 % (312/413) aller Patienten vom Urologen beim Basisbesuch mindestens eine Komorbidität dokumentiert, mit einem signifikanten Unterschied zwischen der Degarelix-Gruppe (76,5 %, 205/268) und der GnRH-Agonistengruppe (73,8 %, 107/145; *p* < 0,0001). Die zu Studienbeginn am häufigsten dokumentierten Begleiterkrankungen (Kodierung gemäß MedDRA) waren für beide Behandlungsgruppen vaskuläre Erkrankungen (einschließlich Hypertonie; 60,3 %, 249/413), gefolgt von Stoffwechsel- und Ernährungserkrankungen (37,0 %, 153/413) und kardialen Erkrankungen (24,2 %, 100/413), wobei in der Degarelix-Gruppe der Anteil der Patienten signifikant höher (*p* < 0,0001) war als in der GnRH-Agonistengruppe (Abb. 1).

Kardiovaskuläre Vorerkrankungen lagen bei 32,2 % (133/413) aller Patienten vor. Herzinsuffizienz (16,0 vs. 11,0 %, *p* < 0,001), periphere arterielle Verschlusskrankheit (pAVK; 12,3 vs. 5,5 %, *p* < 0,0001) in der Anamnese wurden in der Degarelix-Gruppe signifikant häufiger dokumentiert als in der GnRH-Agonistengruppe.

Bei Behandlungsbeginn hatten bereits 15,0 % der Patienten ein schwerwiegendes kardiovaskuläres Ereignis in ihrer Anamnese. 8,6 % der Patienten in der Degarelix-Gruppe hatten bereits einen Myokardinfarkt erlitten im Vergleich zu 7,6 % in der GnRH-Agonistengruppe (*p* < 0,05; Tab. 3).

**Table 3:** 

	Degarelix (*n* = 268)	GnRH-Agonisten (*n* = 145)	Gesamt (*n* = 413)
*Kardiovaskuläre Risikofaktoren*	*192 (71,6* *%)*	*100 (69,0* *%)*^c^	*292 (70,7* *%)*
Hypertonie	159 (59,3 %)	78 (53,8 %)^c^	237 (57,4 %)
Diabetes mellitus	59 (22,0 %)	33 (22,8 %)^a^	92 (22,3 %)
Hyperlipidämie	64 (23,9 %)	28 (19,3 %)^b^	92 (22,3 %)
*Kardiovaskuläre Vorerkrankung*	*90 (33,6* *%)*	*43 (29,7* *%)*^a^	*133 (32,2* *%)*
Herzinsuffizienz	43 (16,0 %)	16 (11,0 %)^b^	59 (14,3 %)
Periphere arterielle Verschlusskrankheit	33 (12,3 %)	8 (5,5 %)^c^	41 (9,9 %)
Koronare Herzkrankheit	8 (3,0 %)	5 (3,4 %)	13 (3,1 %)
*Schweres kardiovaskuläres Ereignis*	*40 (14,9* *%)*	*22 (15,2* *%)*^a^	*62 (15,0* *%)*
Myokardinfarkt	23 (8,6 %)	11 (7,6 %)^a^	34 (8,2 %)
Zerebrovaskuläres Ereignis	19 (7,1 %)	11 (7,6 %)	30 (7,3 %)
Chronische Nierenerkrankung	32 (11,9 %)	6 (4,1 %)^c^	38 (9,2 %)
Chronische Lungenerkrankung	21 (7,8 %)	6 (4,1 %)^a^	27 (6,5 %)
**Familiäre Belastung**			
Kardiovaskuläre Erkrankung bei Verwandten 1. Grades	50 (18,7 %)	26 (17,9 %)^b^	76 (18,4 %)
Diabetes mellitus bei Verwandten 1. Grades	28 (10,4 %)	20 (13,8 %)	48 (11,6 %)
**Lebensstil**			
BMI > 30	51 (19,0 %)	24 (16,6 %)^a^	75 (18,2 %)
Alkoholmissbrauch	5 (1,9 %)	3 (2,1 %)	8 (1,9 %)
Raucher	30 (11,2 %)	10 (6,9 %)^a^	40 (9,7 %)
Ehemaliger Raucher	29 (10,8 %)	23 (15,9 %)	52 (12,6 %)

Mehrfachnennungen möglich

^a^*p* < 0,05, ^b^*p* < 0,001, ^c^p < 0,0001, *p*-Werte basieren auf einem Binomialtest

Kardiovaskuläre Risikofaktoren hatten 70,7 % aller Patienten. In der Degarelix-Gruppe wurden im Vergleich zur GnRH-Agonistengruppe die anamnestischen Risikofaktoren Hypertonie (59,3 vs. 53,8 %, *p* < 0,0001) und Hyperlipidämie (23,9 vs. 19,3 %, *p* = 0,0002) signifikant häufiger dokumentiert (Tab. 3).

Für 81 % (334/413) der Patienten wurde die Begleitmedikation dokumentiert. Die Ergebnisse zeigten einen Unterschied von mindestens 5 % zwischen den Behandlungsgruppen für „Antiandrogene“ (13,4 % Degarelix vs. 39,3 % GnRH-Agonisten; *p* = 0,0000), „Antihypertensiva“ (56,0 % Degarelix vs. 49,0 % Agonisten) und „Antithrombotika/Herztherapie“ (30,2 % Degarelix vs. 24,8 % Agonisten).

### Informationsquelle

**Figure 2:**
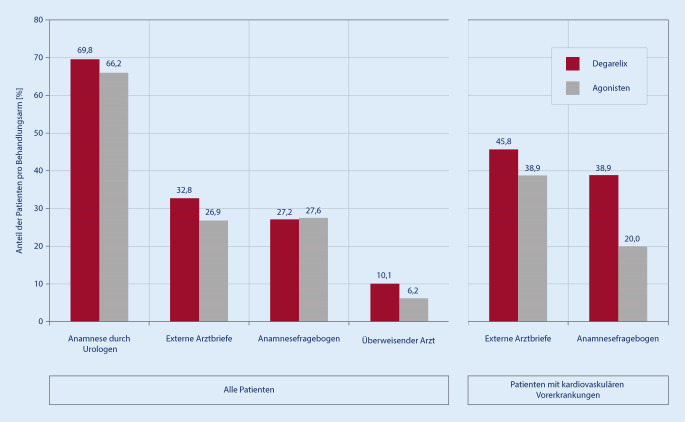

Die Anamnese durch den Urologen war mit 68,5 % die Hauptinformationsquelle für Komorbiditäten mit geringem Unterschied zwischen der Degarelix-Gruppe (69,8 %, 187/268) und der GnRH-Agonistengruppe (66,2 %, 96/145). Bei Patienten mit kardiovaskulären Vorerkrankungen dienten zusätzlich auch der Anamnesefragebogen (Degarelix 38,9 % vs. GnRH-Agonisten 20,0 %) sowie externe Arztbriefe (Degarelix 45,8 % vs. GnRH-Agonisten 38,9 %) als Informationsquelle (Abb. 2).

### Therapieentscheidung

**Figure 3:**
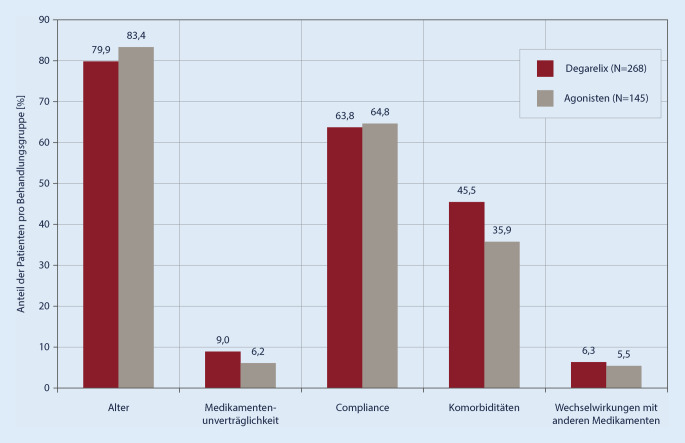

Bei der Erstuntersuchung gaben die Ärzte für 96,1 % der Patienten an, dass die Therapieentscheidung für beide Behandlungsgruppen gleichermaßen einfach war. Ausschlaggebend für die Therapieentscheidung waren für beide Behandlungsgruppen die Faktoren Compliance (64 %), Komorbiditäten (42 %) und Alter (81 %), wobei die Komorbiditäten bei den Degarelix-behandelten Patienten einen prozentual höheren Einfluss auf die Therapieentscheidung hatten, als bei Patienten, die mit einem GnRH-Agonisten behandelt wurden (45,5 vs. 35,9 %, *p* = 0,0577). Von geringerer Bedeutung für die Therapieentscheidung waren Arzneimittelunverträglichkeiten (Degarelix 9,0 % vs. GnRH-Agonisten 6,2 %) oder Wechselwirkungen mit anderen Medikamenten (6,3 vs. 5,5 %) (Abb. 3). Die Analyse der Subgruppen ergab keine statistisch signifikanten Unterschiede.

### Unerwünschte Ereignisse (UE)

Alle innerhalb der Studie dokumentierten UE wurden nach der MedDRA Version 19.0 kodiert. Die UE wurden in nicht schwerwiegende und schwerwiegende UE eingeteilt.

Insgesamt wurden 117 UE während der Behandlungszeit gemeldet, von denen 95 auf die ADT zurückgeführt wurden. Die Rate aller UE betrug 32,1 % (*n* = 86) in der Degarelix-Gruppe und 21,7 % (*n* = 31) in der GnRH-Agonistengruppe. Zu den häufigsten UE, die auf die ADT zurückgeführt wurden, zählten Hitzewallungen (Degarelix 9,3 % vs. GnRH-Agonisten 15,2 %) und Reaktionen an der Injektionsstelle (Degarelix 11,6 % vs. GnRH-Agonisten 0 %). Die Rate schwerwiegender UE betrug 2,2 % (*n* = 6) in der Degarelix-Gruppe und 0 % (*n* = 0) in der GnRH-Agonistengruppe. Folgende schwerwiegende UE traten unter Degarelix-Behandlung auf (Definition nach MedDRA PT): „Apathy, General physical health deterioration, Chills (2 ×), Supraventricular tachycardia, Anemia“*.*

## Diskussion

In der ProComD-Studie konnten Faktoren identifiziert werden, die für die Therapieentscheidung bei Patienten mit hormonnaivem PCa herangezogen werden. Komorbiditäten, Alter und Compliance haben dabei eine hohe Relevanz. Für 76 % der Patienten wurde in dieser Studie mindestens eine Komorbidität dokumentiert. 32 % der Patienten in der ProComD-Studie hatten kardiovaskuläre Vorerkrankungen. Ähnlich hoch (36 %) war der Anteil in einer kürzlich veröffentlichten Versorgungsforschungsstudie [12]. Das durchschnittliche Alter der Patienten lag in der Studie bei 74 Jahren. In einer epidemiologischen Studie in Deutschland lag das durchschnittliche Alter der PCa-Patienten bei 72 Jahren [11].

Die Ergebnisse von zwei großen randomisierten Studien mit PCa-Patienten unter ADT [4, 22] konnten zeigen, dass kardiovaskuläre Ereignisse die zweithäufigste Todesursache (34 bzw. 27 %) nach dem PCa (36 und 41 %) sind. Nach den neuesten EAU-Guidelines übersteigt die kardiovaskuläre Mortalität sogar den Prostatakrebs als häufigste Todesursache [19]. Eine bestehende kardiovaskuläre Erkrankung ist demnach eine der wichtigsten Herausforderungen bei der Behandlung des PCa. Bei der Wahl der ADT sollte die Entscheidung daher auf Präparate fallen, die mit einem geringeren Risiko in Verbindung stehen, diese Begleiterkrankungen zu verstärken [18].

Komorbiditäten spielten bei der Therapieentscheidung für Degarelix eine größere Rolle als bei einer Entscheidung für einen Agonisten (45,5 % Degarelix vs. 35,9 % GnRH-Agonisten). Bei Patienten mit kardiovaskulären Vorerkrankungen wurde Degarelix häufiger eingesetzt als GnRH-Agonisten. Das Patientenkollektiv war in dieser Studie zu klein, um zuverlässige Aussagen bezüglich des Einflusses der ADT auf das kardiovaskuläre Risiko ableiten zu können. Verschiedene Publikationen [2, 3, 15, 23] und aktuelle Reviews [5, 6, 10, 24] deuten jedoch auf die Vorteile der GnRH-Antagonisten bei Patienten mit kardiovaskulären Vorerkrankungen hin.

Die neuen Phase-III-Daten der HERO-Studie zum oralen GnRH-Antagonisten Relugolix bestätigen die Vorteile bei Patienten mit kardiovaskulären Vorerkrankungen. Bei Studieneinschluss hatten 80 % der Patienten mindestens einen kardiovaskulären oder zerebrovaskulären Risikofaktor [21] und 14 % ein schwerwiegendes kardiovaskuläres Ereignis in der Anamnese. Patienten, die mit Relugolix behandelt wurden, hatten ein um 54 % geringeres relatives Risiko für schwerwiegende unerwünschte kardiovaskuläre Ereignisse im Vergleich zu Leuprorelin. In der Subgruppe der Patienten mit einem schwerwiegenden kardiovaskulären Ereignis in der Anamnese war der Unterschied noch deutlicher mit einer 80 %igen Reduktion des relativen Risikos [21].

In einer weiteren kürzlich publizierten prospektiven Phase-II-Studie mit 80 Patienten wurde der Zusammenhang zwischen kardiovaskulären Vorerkrankungen und der Auswirkung der Behandlung mit GnRH-Agonisten gegenüber Degarelix untersucht [16]. Von den Patienten, die einen GnRH-Agonisten erhielten, erlitten in den ersten 12 Monaten 20 % ein schwerwiegendes kardiovaskuläres oder zerebrovaskuläres Ereignis im Vergleich zu 3 % der Degarelix-Patienten (*p* = 0,013), was einer absoluten Risikoreduktion von 18,1 % bzw. einer relativen Risikoreduktion von 88 % entspricht [16]. Die eindringliche Empfehlung der Autoren lautet daher, Patienten mit kardiovaskulären Vorerkrankungen auf diese Daten hinzuweisen, bevor sie sich einer ADT unterziehen.

Eine große Beobachtungsstudie aus Italien mit 9785 Patienten kommt zu einem ähnlichen Ergebnis [20]. Die Inzidenz kardiovaskulärer Ereignisse war signifikant höher bei Patienten, die mit GnRH-Agonisten statt mit Degarelix behandelt wurden (8,8 vs. 6,2, *p* = 0,002). Alter, Bluthochdruck, Dyslipidämie, bestehende kardiovaskuläre Erkrankung und frühere kardiovaskuläre Ereignisse stellten unabhängige Risikofaktoren für ein neues kardiovaskuläres Ereignis dar und wurden in der multivariaten Analyse berücksichtigt. Patienten, die mit Degarelix behandelt wurden, hatten ein geringeres Risiko, ein kardiovaskuläres Ereignis zu erleiden als Patienten unter Agonistentherapie (HR [95%-KI]: 0,76 [0,60–0,95], *p* = 0,018; [20]). Dies wird bestätigt durch die kürzlich publizierten Ergebnisse einer Real-world-Evidenzstudie aus Großbritannien. Das relative Risiko, ein kardiales Ereignis unter ADT zu erleben, war bei Degarelix signifikant geringer als bei GnRH-Agonisten (RR 6,9 vs. 17,7 %; 0,39 [95%-KI 0,191, 0,799]; *p* = 0,01; [8]).

Da Patienten mit kardiovaskulären Risikofaktoren oder Komorbiditäten besonders von einem GnRH-Antagonisten profitieren, steht die Identifizierung dieser im Vordergrund. Eine interdisziplinäre kanadische Arbeitsgruppe gibt Empfehlungen zur Identifizierung von PCa-Patienten, die von einem optimalen Management ihrer kardiovaskulären Erkrankung und/oder einer Änderung der kardialen Risikofaktoren profitieren können. Dies umfasst ein einfaches Screeningtool (STAMP), mit dem Hochrisikopatienten leicht identifiziert werden können [14]. Für die Identifizierung und ggf. Therapie von Patienten mit hohem kardiovaskulärem Risiko existiert auch in Deutschland eine S3-Leitlinie [9].

In einem Kontext, in dem ein besseres Gesamtüberleben ohne Verschlechterung der Lebensqualität eine Herausforderung darstellt, sind die potenziellen kardiovaskulären Nebenwirkungen der ADT von entscheidender Bedeutung. Um die behandlungsbedingten Nebenwirkungen einer ADT patientenindividuell besser einschätzen und bewältigen zu können, müssen Ärzte und Patienten in ständigem Dialog stehen und regelmäßig Informationen austauschen. Da sich in der ProComD-Studie Komorbiditäten als wichtiges Entscheidungskriterium für die ADT herauskristallisiert haben und Degarelix bei Patienten mit kardiovaskulären Vorerkrankungen bevorzugt eingesetzt wurde, besteht die Annahme, dass die bestehenden Kenntnisse den Behandlungsalltag bereits beeinflussen.

In der ProComD-Studie zeigte sich, dass die Anamnese durch den Urologen über beide Arme hinweg als primäre Informationsquelle zu Begleiterkrankungen diente. Besonders bei Patienten mit kardiovaskulären Vorerkrankungen waren außerdem externe Arztbriefe und der Anamnesefragebogen von Bedeutung. Hier wurde deutlich, dass der Anamnesefragebogen bei Patienten mit kardiovaskulären Vorerkrankungen in der Degarelix-Gruppe häufiger als Informationsquelle eingesetzt wurde als in der GnRH-Agonistengruppe.

Die ProComD-Studie unterliegt den üblichen Limitationen einer NIS. Darüber hinaus war keine Mindestdauer der ADT vorgegeben. Dadurch konnten auch Patienten, die nur eine kurzzeitige ADT (z. B. als begleitend adjuvante Therapie im Rahmen einer kurativ intendierten Primärtherapie) erhielten, eingeschlossen werden. Bei diesen spielen evtl. andere Einflussfaktoren bei der Wahl der ADT eine Rolle als bei Patienten, die für eine Langzeitbehandlung intendiert sind.

## Fazit für die Praxis

- Die Faktoren Komorbiditäten, Alter und Compliance beeinflussen die Therapieentscheidung.
- Bestehende kardiovaskuläre Risikofaktoren und Begleiterkrankungen wurden häufiger bei den Degarelix-behandelten Patienten dokumentiert.
- Anamnesefragebögen sowie externe Arztbriefe sind die häufigsten vom Urologen verwendeten Informationsquellen bezüglich vorhandener kardiovaskulärer Begleiterkrankungen.
- Kenntnisse über Komorbiditäten, Risikofaktoren und Begleitmedikation könnten die Therapieentscheidung bezüglich einer Androgendeprivationstherapie (ADT) erleichtern.
